# The burden of metastatic disease of the femur on the Medicare system

**DOI:** 10.1186/s40064-016-3572-8

**Published:** 2016-11-04

**Authors:** Kirollos Gendi, David Hennessy, John Heiner

**Affiliations:** 1 University of Wisconsin School of Medicine and Public Health, 750 Highland Ave, Madison, WI 53726 USA; 2 University of Wisconsin School of Medicine and Public Health, 1685 Highland Ave., UWMFCB-6132, Madison, WI 53705 USA

## Abstract

**Background:**

In the United States, over 1,650,000 new cases of cancer are being diagnosed yearly with almost 50 % of them being the top five bone-seeking cancers. Since cancer risk increases with age, this suggests that orthopedic oncology services may be a strain on the Medicare system. The femur is the most common site of long bone metastases. Prophylactic fixation techniques prevent pathologic fractures, reduce morbidities, and enhance the quality of life of patients with femoral metastases. This study aims to assess the rate of metastatic disease to the skeleton and evaluate the use and financial burden of femoral prophylactic fixation techniques on the Medicare system.

**Questions/purposes:**

(1) In the Medicare population, has the number of skeletal metastases increased? (2) In the Medicare population, has the use of prophylactic fixation techniques increased? (3) How has the financial burden of prophylactic fixation changed over the study period?

**Methods:**

The Medicare database was searched between 2005 and 2014 with the assistance of PearlDiver Technologies Inc. and the RBRVS DataManager Online from the American Medical Association. Searches were completed by using International Classification of Disease-9 (ICD-9) and current procedural terminology (CPT) codes for secondary malignant neoplasms and prophylactic fixation techniques. Facility charges, Medicare reimbursement and length of hospital stay were extracted from the Medicare database. Simple linear regression was performed to test the significance of yearly changes and the coefficient of determination was used to assess the strength of the correlation.

**Results:**

(1) In the Medicare population, has the number of skeletal metastases increased? While the number of Medicare patients with skeletal metastases has increased from 132,452 in 2005 to 155,819 in 2012 (p = 0.01, r^2^ = 0.72), the prevalence of skeletal metastases in this population remained constant at 30.66 cases per 10,000 Medicare patients in 2012 (p = 0.56, r^2^ = 0.06). (2) In the Medicare population, has the use of prophylactic fixation techniques increased? The number of prophylactic fixation techniques has not increased from 2005 to 2014 (p = 0.68, r^2^ = 0.02); however, the rate of prophylactic fixation among those diagnosed with skeletal metastases has significantly decreased from 94.6 per 10,000 in 2005 to 82.72 per 10,000 in 2012 (p = 0.006, r^2^ = 0.74). (3) How has the financial burden of prophylactic fixation changed over the study period? Both total and average hospital charges increased after adjusting for inflation in the total Medicare population; however, only the average Medicare reimbursement changed to reflect this. The total amount Medicare spent on prophylactic fixation techniques in 2012 was $20,245,957 after adjusting to 2014. Despite the increase in hospital charges and average Medicare reimbursement, the average length of hospital stay in the total Medicare population showed a significant decreased trend—down from 7.51 days in 2005 to 5.86 days in 2012 (p = 0.02, r^2^ = 0.81).

**Conclusions:**

Although the prevalence of metastatic disease to the skeleton remained stable between 2005 and 2012 in the Medicare population, prophylactic femoral fixation techniques declined in elderly adults between 2005 and 2014. This most likely signifies an increase in other treatment modalities that can prevent pathologic fractures such as prophylactic hemiarthroplasty, bisphosphonates, and/or radiation therapy.

**Level of evidence:**

Level IV, Cross-sectional Study.

## Introduction

### Background

In the United States, over 1,650,000 new cases of cancer are diagnosed every year and almost 50 % of these cases are the top five bone-seeking cancers: multiple myeloma, breast, lung, prostate and kidney (American Cancer Society 2012; Kelly et al. 2012). As a group, these cancers have shown an increase in the number of cases diagnosed each year for at least the past decade (American Cancer Society 2005, 2006, 2007, 2008, 2009, 2010, 2011, 2012). Of cancers that metastasize to the skeleton, the most common site in long bones is the femur (Hattori et al. 2007; Toliusis et al. 2010). Metastatic disease of the femur is associated with severe pain, functional decline, and pathologic fracture, which can lead to a significantly worse quality of life and a state of dependency (Bickels et al. 2009). Given the devastating consequences of pathologic femur fractures, orthopedic oncologists use prophylactic fixation techniques to improve their patients’ pain, mobility, and quality of life (Alvi and Damron 2013; Arvinius et al. 2014; Bickels et al. 2009; Gartrell and Saad 2014; Haidukewych 2012; Hattori et al. 2007; Miller et al. 2011; Moon et al. 2014; Ristevski et al. 2009; Toliusis et al. 2010). Other benefits of prophylactic fixation of an impending fracture include decreased hospital stay, earlier return to ambulation, and improved survival (Arvinius et al. 2014; Haidukewych 2012; Ristevski et al. 2009; Toliusis et al. 2010). Intramedullary nails and cephalomedullary nails are widely used as means to treat impending pathologic fractures. These fixation methods aim to maximize the remaining quality of life as over 60 % of patients with metastatic disease of the femur that require intervention are likely to die within the year (Miller et al. 2011; Ristevski et al. 2009).

### Rationale

Since cancer diagnoses increase with age, this suggests that the amount of orthopedic oncology services and their associated costs could be a strain on the Medicare system. Given the potentially high demand for these orthopedic procedures to treat metastatic disease of the femur, this study aims to assess the patient load and financial burden associated with prophylactic fixation within the Medicare inpatient population from 2005 to 2014. We hypothesize that, as a product of the increasing burden of metastatic disease, there has been an increase in the utilization of prophylactic fixation and associated healthcare costs.

### Study questions

1. In the Medicare population, has the number of skeletal metastases increased?
2. In the Medicare population, has the use of prophylactic fixation techniques increased?
3. How has the financial burden of prophylactic fixation changed over the study period?

## Methods

### Study design and setting

This is a retrospective, cross-sectional study of the Medicare database between 2005 and 2014.

### Participants/study subjects

This study looked at the Medicare inpatient database from 2005 to 2014. Medicare patients who underwent prophylactic fixation of the femur between 2005 and 2012 were identified through PearlDiver Technologies Inc., which specializes in data retrieval for orthopedic patients through current procedural terminology (CPT) codes and International Classification of Disease-9 (ICD-9) codes. This was the most up-to-date information that PearlDiver possessed due to the time it takes to compile the financial information. Additionally, CPT codes were used to search the Medicare database between 2013 and 2014 with the Resource-Based Relative Value Scale (RBRVS) DataManager Online from the American Medical Association (RBRVS DataManager Online 1995).

### Description of experiment, treatment or surgery

Patients in the study were identified using ICD-9 inpatient procedural code 78.55, which encompasses CPT 27187, “prophylactic treatment (nailing, pinning, plating or wiring) with or without methylmethacrylate, femoral neck and proximal femur,” and CPT27495, “prophylactic treatment (nailing, pinning, plating, or wiring) with or without methylmethacrylate, femur.” This was crossed with ICD-9 198.5, the code for secondary malignant neoplasm of the bone or bone marrow. ICD-9 198.5 was also run on its own by PearlDiver during this time period to see if the burden of metastatic disease to the skeleton has been increasing. Separately, CPT 27495 and 27187 were used to search the Medicare database between 2013 and 2014 through the RBRVS (RBRVS DataManager Online 1995).

### Variables, outcome measures, data sources, and bias

Prevalence was calculated using enrollment data from the Centers for Medicare and Medicaid Services 2013 Statistical Supplement (Centers for Medicare and Medicaid Services 2013). Since this information did not include gender sub-groups, these were omitted when calculating prevalence. All patient information was de-identified by PearlDiver, so the study did not need action through an Institutional Review Board. Facility charges, Medicare reimbursement, gender, age, and length of hospital stay were extracted from the Medicare database by PearlDiver.

### Statistical analysis, study size

Linear regression was performed to test the significance of yearly changes in the number of procedures, number of patients coded for skeletal metastases, Medicare reimbursement, hospital charges, and length of hospital stay. A significant upward trend is reported with a p value less than 0.05, the strength of the correlation is indicated by the coefficient of determination, r^2^, and the regression coefficients with their 95 % confidence intervals have also been calculated. All charges were adjusted for inflation to the year 2014 (Adjust for inflation 2014; Statistics USBoL 2014; US Inflation Calculator 2014).

## Results

### (1) In the Medicare population, has the number of skeletal metastases increased?

The results of our search were separated into seven different groups based on age above or below 65 and gender. Patients under 65 can qualify for Medicare if they have been diagnosed with end stage renal disease (ESRD) or amyotrophic lateral sclerosis (ALS). Additionally, they may qualify if they have been on social security disability income (SSDI) for 2 years (Signing up for Medicare 2016). These patients may be able to dual qualify for both Medicare and Medicaid, but the data in this study only represents charges to the Medicare system.

**Fig. 1:**
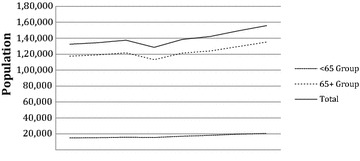
Medicare population diagnosed with skeletal metastases

The Medicare population that had been diagnosed with metastatic disease to the skeleton are displayed in Table 1 and Fig. 1. In the most recent year, there were a total of 155,819 patients in the Medicare system coded for metastatic disease to the skeleton. All seven groups had significant upward trends and strong correlations between 2005 and 2012. Additionally, the number of diagnoses increased by an average of 3124 (1176; 5073) each year during the study period. Despite this, as shown in Table 2, the prevalence of skeletal metastases in the Medicare population remained stable with a rate of 30.66 per 10,000 (p = 0.56, r^2^ = 0.058) in 2012. This averaged to a yearly change of −0.0948 (−0.48; 0.29).

**Table 1:** Medicare inpatient population diagnosed with metastatic disease to the skeleton

Year	Men <65	Women <65	<65 group	Men 65+	Women 65+	65+ group	Total
2005	5762	9206	14,968	64,638	52,846	117,484	132,452
2006	6008	9299	15,307	65,715	53,342	119,057	134,364
2007	6261	9540	15,801	67,650	54,047	121,697	137,498
2008	6103	9388	15,491	62,618	50,465	113,083	128,574
2009	6824	10,302	17,126	67,512	53,843	121,355	138,481
2010	7208	10,952	18,160	68,933	55,123	124,056	142,216
2011	7931	11,776	19,707	72,878	56,741	129,619	149,326
2012	8195	12,410	20,605	76,251	58,963	135,214	155,819
r^2^	*0.93*	*0.89*	*0.91*	*0.68*	*0.57*	*0.64*	*0.72*
p value	*0.0001*	*0.0004*	*0.0002*	*0.01*	*0.03*	*0.02*	*0.01*
Co (LCL; UCL)	*360 (257; 462)*	*476 (312; 640)*	*835 (572; 1099)*	*1498 (479; 2517)*	*791 (101; 1480)*	*2289 (586; 3992)*	*3124 (1176; 5073)*

Italic values indicate statistically significance at p < 0.05

**Table 2:** Prevalence of skeletal metastases in the Medicare population per 10,000

Year	<65 group	65+ group	Total
2005	22.26	32.84	31.17
2006	21.80	32.78	31.00
2007	21.65	32.92	31.06
2008	20.61	29.84	28.31
2009	22.08	31.30	29.77
2010	22.61	31.30	29.84
2011	23.53	32.03	30.57
2012	23.89	32.04	30.66
r^2^	0.46	0.13	0.06
p value	0.065	0.39	0.56
Co (LCL; UCL)	0.29 (−0.02; 0.61)	−0.15 (−0.55; 0.25)	−0.0948 (−0.48; 0.29)

### (2) In the Medicare population, has the use of prophylactic fixation techniques increased?

**Fig. 2:**
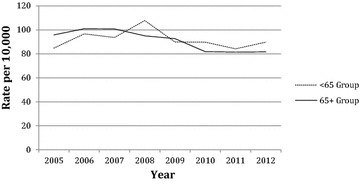
Rates of prophylactic femoral fixation

The population that underwent prophylactic fixation of their femurs is detailed in Table 3. A total of 1364 prophylactic fixation procedures were coded for in 2014. Overall, there was no upward trend in the total population (p = 0.68, r^2^ = 0.02) or the over 65 age group (p = 0.11, r^2^ = 0.36). These corresponded to an average yearly change of 3.41 (−15.20; 22.01) and −17.50 (−40.70; 5.70), respectively. When further subdivided, the less than 65 age group and the women less than 65 age group did show significant positive trends, (p = 0.003, r^2^ = 0.80) and (p = 0.001, r^2^ = 0.84), respectively. The yearly changes for these values were 6.29 (3.16; 9.41) and 5.18 (2.89; 7.46), respectively. Table 4 and Fig. 2 show the rate of femoral prophylactic fixation procedures among those with skeletal metastases per 10,000 Medicare patients. There is no trend in the less than 65 age group (p = 0.60, r^2^ = 0.05), which had an average yearly change of −0.68 (−3.69; 2.32). However, there is a significant decreased trend in the over 65 age group (p = 0.004, r^2^ = 0.78), which changed by −3.05 (−4.67; −1.43) each year during the study period.

**Table 3:** Medicare inpatient population that had their femurs prophylactically fixated

Year	Men <65	Women <65	<65 group	Men 65+	Women 65+	65+ group	Total
2005	47	80	127	507	619	1126	1253
2006	59	89	148	515	687	1202	1350
2007	57	91	148	538	689	1227	1375
2008	61	106	167	490	585	1075	1242
2009	57	97	154	474	651	1125	1279
2010	48	115	163	467	548	1015	1178
2011	60	106	166	478	578	1056	1222
2012	64	121	185	491	613	1104	1289
2013^a^	–	–	–	–	–	–	1372
2014^a^	–	–	–	–	–	–	1364
r^2^	0.20	*0.84*	*0.80*	0.42	0.29	0.36	0.02
p value	0.27	*0.001*	*0.003*	0.08	0.17	0.11	0.68
Co (LCL; UCL)	1.11 (−1.11; 3.32)	*5.18 (2.89; 7.46)*	*6.29 (3.16; 9.41)*	−6.26 (−13.66; 1.13)	−11.24 (−28.89; 6.42)	−17.50 (−40.70; 5.70)	3.41 (−15.20; 22.01)

^a^Data collected from CPT codes from the Resource-Based Relative Value Scale (RBRVS) DataManager Online from the American Medical Association

Italic values indicate statistically significance at p < 0.05

**Table 4:** Rate of prophylactic fixation in the Medicare population with skeletal metastases per 10,000

Year	<65 group	65+ group	Total
2005	84.85	95.84	94.60
2006	96.69	100.96	100.47
2007	93.67	100.82	100.00
2008	107.80	95.06	96.60
2009	89.92	92.70	92.36
2010	89.76	81.82	82.83
2011	84.23	81.47	81.83
2012	89.78	81.65	82.72
r^2^	0.05	*0.78*	*0.74*
p value	0.60	*0.004*	*0.006*
Co (LCL; UCL)	−0.68 (−3.69; 2.32)	*−3.05 (−4.67; −1.43)*	*−2.76 (−4.41; −1.12)*

Italic values indicate statistically significance at p < 0.05

### (3) How has the financial burden of prophylactic fixation changed over the study period?

**Fig. 3:**
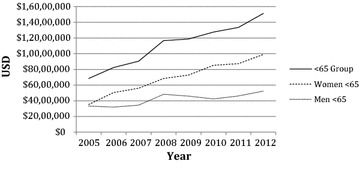
Estimated total hospital charges

The estimated total hospital charges for the Medicare inpatient population that had their femurs prophylactically fixated is displayed in Table 5 while the average estimated hospital charge is displayed in Table 6. In 2012, the estimated total charge for ICD-9 78.55 was $100,951,048 while the estimated average hospital charge was $78,317 per case after adjusting for inflation to 2014. The upward trend in the total charge was significant for the total population with the regression coefficient indicating that it increased on average by $3,274,409 ($1,022,310; $5,526,507) each year. This significant upward trend was echoed in the less than 65 age groups, which is displayed in Fig. 3. However, it was not significant in the groups above the age of 65. Moreover, the estimated average charge for the total population was significant (p = 0.0007, r^2^ = 0.87) and increased by an average of $3104 ($1921; $4287) between 2005 and 2012. All groups were shown to have significant upward trends for average hospital charges except for the men below age 65 (p = 0.05, r^2^ = 0.49).

**Table 5:** Estimated total hospital charges for the Medicare inpatient population coded for 78.55 after adjusting for inflation to 2014

Year	Men <65	Women <65	<65 group	Men 65+	Women 65+	65+ group	Total
2005	$3,321,798	$3,522,457	$6,844,255	$28,106,043	$33,916,448	$62,022,491	$68,866,746
2006	$3,191,238	$5,046,752	$8,237,990	$28,166,564	$36,474,070	$64,640,634	$72,878,624
2007	$3,440,358	$5,597,546	$9,037,904	$32,380,628	$39,863,264	$72,243,892	$81,281,796
2008	$4,824,453	$6,835,547	$11,660,000	$28,677,566	$33,188,385	$61,865,951	$73,525,952
2009	$4,594,260	$7,274,126	$11,868,386	$30,259,296	$39,484,025	$69,743,321	$81,611,707
2010	$4,234,515	$8,517,251	$12,751,766	$31,855,697	$37,268,545	$69,124,242	$81,876,810
2011	$4,613,752	$8,731,962	$13,345,714	$27,523,401	$40,127,393	$67,650,794	$80,996,508
2012	$5,229,775	$9,900,519	$15,130,294	$42,865,171	$42,955,385	$85,820,556	$100,951,048
r^2^	*0.74*	*0.98*	*0.97*	0.34	0.49	0.47	*0.68*
p value	*0.006*	*1.76103E−6*	*0.00001*	0.13	0.05	0.06	*0.01*
Co (LCL; UCL)	*269,294 (107,962; 430,626)*	*860,359 (744,807; 975,912)*	*1,129,653 (918,534; 1,340,773)*	1,191,726 (−481,137; 2,864,589)	952,984 (−12,553; 1,918,521)	2,144,710 (−147,181; 4,436,601)	*3,274,409 (1,022,310; 5,526,507)*

Italic values indicate statistically significance at p < 0.05

**Table 6:** Estimated average hospital charge for the Medicare inpatient population coded for 78.55 after adjusting for inflation to 2014

Year	Men <65	Women <65	<65 group	Men 65+	Women 65+	65+ group	Total
2005	$70,677	$44,031	$53,892	$55,436	$54,792	$55,082	$54,961
2006	$54,089	$56,705	$55,662	$54,692	$53,092	$53,778	$53,984
2007	$60,357	$61,511	$61,067	$60,187	$57,857	$58,878	$59,114
2008	$79,089	$64,486	$69,820	$58,526	$56,732	$57,550	$59,200
2009	$80,601	$74,991	$77,067	$63,838	$60,651	$61,994	$63,809
2010	$88,219	$74,063	$78,232	$68,213	$68,008	$68,103	$69,504
2011	$76,896	$82,377	$80,396	$57,580	$69,425	$64,063	$66,282
2012	$81,715	$81,822	$81,785	$87,302	$70,074	$77,736	$78,317
r^2^	0.49	*0.94*	*0.93*	*0.53*	*0.89*	*0.80*	*0.87*
p value	0.05	*0.00009*	*0.00009*	*0.04*	*0.0004*	*0.003*	*0.0007*
Co (LCL; UCL)	3291 (−81; 6662)	*5251 (3872; 6629)*	*4496 (3302; 5690)*	*3177 (182; 6173)*	*2655 (1745; 3564)*	*2882 (1461; 4304)*	*3104 (1921; 4287)*

Italic values indicate statistically significance at p < 0.05

**Fig. 4:**
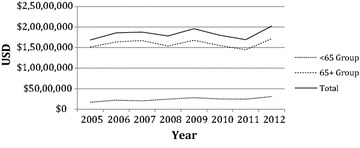
Total Medicare reimbursement

The estimated total Medicare reimbursement for ICD-9 78.55 is detailed in Table 7. Overall, the Medicare system paid $20,245,957 for prophylactic fixation procedures in 2012 after adjusting for inflation to 2014. However, there was no significant upward trend in the total population, the above 65 age groups, or men under 65. This is illustrated in Fig. 4. Only the combined less than 65 age group and the women less than 65 age group showed significant upward trends in their estimated total reimbursement, (p = 0.007, r^2^ = 0.73) and (p = 0.003, r^2^ = 0.79), respectively. The estimated average Medicare reimbursement per case is shown in Table 8. In 2012, the average Medicare reimbursement for the total population was $15,707 per case after adjusting for inflation to 2014. This showed a significant positive trend (p = 0.04, r^2^ = 0.53) and the reimbursement increased by an average of $263 ($15; $510) per year between 2005 and 2012 in the total population. Similarly, the women over 65 age group displayed a significant upward trend (p = 0.04, r^2^ = 0.53), as did the combined over 65 age group (p = 0.04, r^2^ = 0.52). However, the men over 65 age group and the less than 65 age groups did not display significant trends.

**Table 7:** Estimated total Medicare reimbursement for the Medicare inpatient population coded for 78.55 after adjusting for inflation to 2014

Year	Men <65	Women <65	<65 group	Men 65+	Women 65+	65+ group	Total
2005	$721,491	$995,625	$1,717,115	$6,864,726	$8,274,647	$15,139,373	$16,856,488
2006	$973,424	$1,231,249	$2,204,673	$6,811,380	$9,572,709	$16,384,089	$18,588,762
2007	$804,462	$1,245,873	$2,050,335	$7,364,483	$9,348,865	$16,713,348	$18,763,683
2008	$992,739	$1,466,225	$2,458,964	$7,257,886	$8,118,277	$15,376,163	$17,835,127
2009	$1,067,505	$1,741,063	$2,808,569	$7,119,188	$9,660,526	$16,779,715	$19,588,283
2010	$777,511	$1,717,583	$2,495,094	$7,194,923	$8,308,407	$15,503,330	$17,998,424
2011	$989,193	$1,475,928	$2,465,122	$6,372,876	$8,091,785	$14,464,661	$16,929,783
2012	$1,070,240	$2,013,565	$3,083,805	$8,045,193	$9,116,958	$17,162,151	$20,245,957
r^2^	0.29	*0.79*	*0.73*	0.11	0.02	0.005	0.13,
p value	0.17	*0.003*	*0.007*	0.43	0.75	0.87	0.38,
Co (LCL; UCL)	29,929 (−16,960; 76,817)	*119,551 (57,730; 181,293)*	*149,440 (59,667; 239,213)*	64,564 (−123,165; 252,293)	−36,757 (−311,001; 237,487)	27,807 (−358,709; 414,323)	177,247 (−276,695; 631,190)

Italic values indicate statistically significance at p < 0.05

**Table 8:** Estimated average Medicare reimbursement for the Medicare inpatient population coded for 78.55 after adjusting for inflation to 2014

Year	Men <65	Women <65	<65 group	Men 65+	Women 65+	65+ group	Total
2005	$15,351	$12,445	$13,521	$13,540	$13,368	$13,040	$13,453
2006	$16,499	$13,834	$14,896	$13,226	$13,934	$13,219	$13,769
2007	$14,113	$13,691	$13,854	$13,689	$13,569	$13,210	$13,646
2008	$16,274	$13,832	$14,724	$14,812	$13,877	$13,872	$14,360
2009	$18,728	$17,949	$18,237	$15,019	$14,840	$14,465	$15,315
2010	$16,198	$14,936	$15,307	$15,407	$15,161	$14,813	$15,279
2011	$16,487	$13,924	$14,850	$13,332	$14,000	$13,284	$13,854
2012	$16,722	$16,641	$16,669	$16,385	$14,873	$15,076	$15,707
r^2^	0.17	0.37	0.32	0.43	*0.53*	*0.52*	*0.53*
p value	0.31	0.11	0.14	0.08	*0.04*	*0.04*	*0.04*
Co (LCL; UCL)	217 (−267; 701)	448 (−131; 1,028)	343 (−158; 843)	307 (−48; 662)	*198 (12; 383)*	*353 (−162; 869)*	*263 (15; 510)*

Italic values indicate statistically significance at p < 0.05

Moreover, the estimated length of hospital stay for the Medicare inpatient population coded for ICD-9 78.55 is shown in Table 9. The total population showed a significant decline in the length of hospital stay from 7.51 days in 2005 to 5.86 days in 2012 (p = 0.02, r^2^ = 0.81). This represents an average decline of 0.22 (0.33; 0.11) days each year. However, three groups did not have significant declines in length of hospital stay: men less than 65 (p = 0.13, r^2^ = 0.35), women less than 65 (p = 0.69, r^2^ = 0.03), and men over 65 (p = 0.28, r^2^ = 0.19). Despite this, the combined less than 65 age group did show a significant downward trend (p = 0.02, r^2^ = 0.61) with an average decline of −0.15 (−0.27; −0.03) days each year.

**Table 9:** Estimated average length of hospital stay during a prophylactic fixation procedure

Year	Men <65	Women <65	<65 group	Men 65+	Women 65+	65+ group	Total
2005	9	6	7.11	7	8	7.55	7.51
2006	7	7	7	7	7	7	7
2007	7	7	7	7	7	7	7
2008	8	6	6.73	6	6	6	6.1
2009	7	6	6.37	6	6	6	6.04
2010	9	6	6.88	6	6	6	6.12
2011	6	7	6.64	6	6	6	6.09
2012	5	6	5.65	7	5	5.89	5.86
r^2^	0.35	0.03	*0.61*	0.19	*0.85*	*0.78*	*0.81*
p value	0.13	0.69	*0.02*	0.28	*0.001*	*0.004*	*0.02*
Co (LCL; UCL)	−0.33 (−0.79; 0.12)	−0.04 (−0.24; 0.17)	*−0.15 (−0.27; −0.03*	−0.10 (−0.29; 0.10)	*−0.35 (−0.49; −0.20)*	*−0.23 (−0.36; −0.11)*	*−0.22 (−0.33; −0.11)*

Italic values indicate statistically significance at p < 0.05

## Discussion

### Background and rationale

As the number of cases of bone-seeking cancers increases in the United States, the amount of orthopedic oncology services and their associated costs could be a strain on the Medicare system. This study was completed to evaluate the burden of femoral metastases on the Medicare system and to analyze the associated charges.

### Limitations

This study had a number of limitations. First, this is a retrospective observational study that relied on the Medicare inpatient database. All such databases are subject to errors and inaccuracies in coding. For instance, while a prophylactic intramedullary nailing should be coded with 27495, it may have been miscoded with CPT 27506: “Open treatment of femoral shaft fracture, with or without external fixation, with insertion of intramedullary implant, with or without curettage.” Second, the database was not designed to provide more specific patient information such as age, comorbidities, preoperative diagnoses, or postoperative diagnoses, so this limited our ability to gather more detailed information on the population with femoral metastases.

Third, no ICD-9 code that is specific to metastatic disease of the femur exists, so the best surrogate marker was ICD-9 code 198.5—secondary malignant neoplasm of bone or bone marrow. Due to this, we had no way of knowing the percentage of patients with metastatic disease of the femur that did not undergo prophylactic surgical management. However, it is reasonable to assume that the percentage is increasing in the over 65 age groups since the utilization of prophylactic fixation techniques has not increased, but the number of cases of metastatic disease to the skeleton has increased. Fourth, ICD-9 code 78.55 encompasses prophylactic fixation techniques for metastatic disease to the femur, so we were unable to study trends in specific procedures. While more specific CPT codes do exist, these are not used for coding the inpatient Medicare population in the PearlDiver database. Additionally, the data that PearlDiver has available is a couple of years behind the current date because of all the data they compile. In order to get more current information, we used the RBRVS DataManager Online from the American Medical Association to gather data on prophylactic femoral fixation in the years 2013–2014. Due to inaccuracies with coding, CPT 27495 and 27187 may not match the results of ICD-9 78.55 perfectly. Also, we could only extract total volume for these procedures—we did not have access to information regarding hospital charges, Medicare reimbursement, or length of hospital stay.

Moreover, during the statistical analysis, an emphasis on the significance of the trends was placed. This, combined with multiple sub-group analyses, increased our chances of a false positive result.

Finally, since the data we gathered from the Medicare database lags by a couple of years, there is time for trends to change between what the database reflected and what is currently happening in clinical practice.

### Discussion: (1) In the Medicare population, has the number of skeletal metastases increased?

The data we collected confirms that there is an increased burden of skeletal metastases in the Medicare population since all groups showed significant upward trends between 2005 and 2012. While the increase in cases does burden the Medicare system, the prevalence of skeletal metastases among Medicare patients has not changed. This reflects the increase in cases of bone seeking cancers, which is what data from the American Cancer Society suggests (American Cancer Society 2005, 2006, 2007, 2008, 2009, 2010, 2011, 2012). Also, the increased number of cases can be explained by the increase in the Medicare population (Centers for Medicare and Medicaid Services 2013).

### Discussion: (2) In the Medicare population, has the use of prophylactic fixation techniques increased?

#### Utilization of prophylactic femoral fixation in adults over age 65

With the increased cases of skeletal metastases, the potential need for surgical management of metastatic disease to the skeleton should be increasing. After focusing our attention on the most common site of long bone metastases, we found that prophylactic fixation of the femur, as reported by ICD-9 inpatient procedural code 78.55, CPT 27495, and CPT 27187, has not shown a significant increase in the total Medicare population between 2005 and 2014. Also, the largest of the Medicare subpopulations, adults over the age of 65, did not show an increasing trend between 2005 and 2012. In fact, its rate of use among older adults with skeletal metastases significantly decreased.

Since prophylactic fixation procedures have decreased despite the consistent prevalence of metastatic disease to the skeleton, then there are either fewer metastases to the femur, there are other CPT codes being used, or there are other treatment modalities that are increasingly being utilized to prevent impending fractures. Despite our inability to specifically study the rate of femoral metastases because no ICD-9 code for it exists, we believe that the notion that femoral metastases are decreasing can be dismissed. There is simply no evidence that cancer pathology or therapy have changed in a way that would alter the location preference of a bone-seeking cancer.

Another possible explanation is that prophylactic fixation techniques have not increased because other procedures are being used to stabilize impending fractures. The use of CPT code 27125, “hemiarthroplasty, hip, partial (e.g., femoral stem prosthesis, bipolar arthroplasty)” could explain why prophylactic fixation techniques have not become more common. Studying the rate of CPT 27125 could be an avenue of future research; however, it may be difficult to know whether the procedures were done prophylactically since it is not specified in the code and no prophylactic hemiarthroplasty code exists.

The third explanation, other treatment modalities are stabilizing femoral metastases, is the most plausible. In particular, radiotherapy and osteoclast inhibiting medications have been used to treat metastatic disease to the femur (Bickels et al. 2009). Radiation has been effective for pain management in those with metastatic femoral lesions and was shown to circumvent the need for surgical intervention in 81 % of impending fracture cases in one study (Harada et al. 2010). Other benefits of using radiation therapy over surgery include decreased pain, risk of DVT, fat embolism, anesthesia risk, and hospital stay (Hattori et al. 2007; Kelly et al. 2012; Swanson et al. 2000). Additionally, using radiotherapy instead of surgery may be more cost effective than prophylactic fixation and further studies would be required to evaluate this.

Another non-surgical treatment is the use of bisphosphonates or RANKL inhibitors, which prevent osteoclasts from resorbing bone. They have both been shown to decrease the number of bony metastases, decrease the prevalence of pathologic fractures, and prevent the need for surgical fixation (Bickels et al. 2009; Gartrell and Saad 2014; Saad et al. 2002). While both bisphosphonates and RANKL inhibitors are effective, a recent meta analysis has shown that RANKL inhibitors are better at preventing pathologic fractures than bisphosphonates (Lipton et al. 2012). The increased use of these medications may be circumventing the need for prophylactic fixation. Again, comparing the costs of this therapy with the costs of prophylactic fixation could be a future area of research to determine the most effective use of resources.

Despite a possible increase in the utilization of other treatment modalities, the benefits of early prophylactic fixation have recently been re-emphasized in the literature. One study did so by comparing prophylactic intramedullary nailing outcomes with the outcomes of therapeutic intramedullary nailing after a pathologic fracture. Significant benefits supporting the use of prophylactic nailing included shorter hospital stay, earlier weight bearing, and increased survival (Arvinius et al. 2014). Also, it is important to note that the majority of pathologic fractures never heal, especially if they have previously received radiation therapy (Haidukewych 2012; Miller et al. 2011). This could adversely affect ambulation and put the hardware at a higher risk of failure.

Additionally, prophylactic intramedullary nails have been shown to have a low failure rate, 11 %, and a low complication rate, 12.5 %, which support their continued use (Alvi and Damron 2013). This has led to the conclusion that it is appropriate to protect the entire length of the bone in case of disease progression. However, one study suggests that it is unnecessary to routinely protect the femoral neck since none of their 145 study participants developed metastases in that region (Moon et al. 2014).

#### Utilization of prophylactic femoral fixation in the under age 65 population

The two groups that did show significant upward trends in the use of prophylactic fixation techniques were total adults under the age of 65 and women under the age of 65. Since men under the age of 65 did not show a significant trend, it is likely that the women in the total population under 65 were the driving force for its significance.

Despite the increase in the number of prophylactic fixation procedures, the rate of their use among those with skeletal metastases did not change in the less than 65 group. It stayed consistent with the prevalence of skeletal metastases. This contrasts with the older population, which saw a decreased trend in the context of an unchanging prevalence.

There has been some evidence that younger women, particularly those less than age 35, have worse breast cancer prognoses (Fredholm et al. 2009; Liu et al. 2015). Since they have more aggressive tumors, perhaps they did not respond well enough to radiotherapy or osteoclast inhibitors and required surgical fixation more often. Also, since they were younger, surgery may have been used more readily since their potential for survival may have been greater or overestimated.

### Discussion: (3) How has the financial burden of prophylactic fixation changed over the study period?

#### Increasing total hospital charges in the under 65 age group

When looking at the estimated total charges, both the overall population’s total hospital charges and the less than 65 age groups had significant upward trends. Because the over 65 age groups did not have significant trends, it is likely that the under 65 age groups were the drivers of the total group’s trend.

A possible explanation for why the younger groups had higher hospital charges could be that those who qualify for Medicare under the age of 65 have significant comorbidities that could be increasing their cost of care.

#### Significant increase in average hospital charges, but not total hospital charges

All groups except for men under age 65 showed a significant upward trend in the average hospital charges for ICD-9 78.55. It seems contradictory that the total hospital charge for the over 65 age group was not statistically significant, but the average hospital charge was statistically significant. An explanation for this is that the number of procedures has not increased, but the cost per procedure has. This would reflect a higher average hospital charge that may not be reflected in the total hospital charge.

This requires an explanation of why the average charge is increasing after adjusting for inflation. Since there are other treatment modalities in place to treat metastatic disease of the femur, surgery may be seen as more of a final effort to prevent a pathologic fracture. Studies on some surgical procedures, such as pancreaticoduodenectomy, have shown that a decreased volume of procedures can lead to an increase in its cost (Sutton et al. 2014). The decreased use of ICD-9 78.55 could therefore be increasing its value.

While this explanation holds true for the above 65 age groups since they did not have an increase in prophylactic fixation, the less than 65 age group and the women less than 65 age group did have a significant upward trend in prophylactic fixation, so the explanation of fewer procedures yields a higher cost does not apply here. As mentioned previously, the less than age 65 population may have significant comorbidities, which allowed them to enroll in Medicare. The management of those conditions could also be contributing to their increased average hospital charge. For instance, ESRD is one condition that qualifies the under 65 age group for Medicare. Hemodialysis is known to be an expensive procedure and if the patient required dialysis during their hospitalization for prophylactic femoral fixation then this would increase the hospital charge during their stay.

#### Medicare reimbursement rates

When looking at the total Medicare inpatient reimbursements, only the less than 65 combined age group and the women less than 65 age group showed a significant increasing trend. This reflects the increasing total hospital charges for these groups. Although the men less than 65 age group and the total Medicare inpatient group showed significantly increasing total hospital charges, this was not reflected in their total Medicare reimbursement. The reasons for how Medicare decides their reimbursement rate is beyond the scope of this paper, but our data suggests that it is not solely a proportion of the amount the hospitals decide to charge.

Lastly, the average Medicare reimbursement for ICD-9 78.55 showed a significant upward trend in the women over 65 age group, the combined over 65 age group, and the total Medicare inpatient population. However, all of the significant values were on the lower ends of what is considered a strong correlation and the combined over 65 age group and the total group likely owe their significance to the women over 65 age group. Despite showing a significant increased trend in average hospital charge, the women less than 65 age group, the combined less than 65 age group, and the men over 65 age group did not have a significant positive trend in their average Medicare reimbursement. Again, this suggests that Medicare reimbursement is not simply a proportion of what the hospitals charge.

#### Length of hospital stay

Even though total and average hospital charges have increased in the total Medicare population, both the over and under 65 year old combined groups, the women over 65 age group, and the total population group showed a significant decreased trend in the average hospital stay. However, since both the men less than 65 and the women less than 65 groups did not show a significant decreased trend, it is possible that the significant trend in the combined less than 65 group is an artifact. The women less than 65 group essentially shows no change in their average hospital stay while the men less than 65 start high at 9 days then drop down to only 6 and 5 days in the last 2 years. This decrease in the last 2 years happened too late in the study period to affect the overall trend in this group, but when averaged with the women under 65 in the combined less than 65 age group, it was able to form a trend.

With regard to the over 65 age group, the decreased trend in average hospital stay was likely driven by the women over 65 group. Since the population in this group was so large, it may have also driven the decreased trend in the total group as well.

## Conclusions

The benefits of prophylactically fixating femoral metastases are well documented and, since the number of bone-seeking cancers is on the rise in the US, the financial burden that a high volume of the procedure places on the Medicare system could be staggering. This study confirms the increased burden of metastatic disease to the skeleton in all of our Medicare subpopulations even though its prevalence remained stable. Despite this, the rate of prophylactic fixation techniques decreased between 2005 and 2012 in the largest Medicare subpopulation, adults over 65. This may reflect the increased utilization of prophylactic hemiarthroplasty or non-surgical therapy. Future studies comparing ICD-9 inpatient procedural code 78.55 to these therapies would need to be conducted to affirm this.

Although adults over 65 have not shown an increase in prophylactic femoral fixation procedures, women under 65 did have a significant increased trend and a consistent rate. This may be due to the presence of more aggressive breast cancers in younger women that do not respond to non-surgical therapy and an increased readiness of clinicians to recommend surgical fixation to a younger population.

From a financial perspective, Medicare spent $20,245,957 in 2012 on reimbursements for ICD-9 inpatient procedural code 78.55 after adjusting for inflation to 2014. While this is widely considered to be a cost-saving procedure, future studies should compare the cost effectiveness of it with the treatment modalities that are potentially replacing it. Depending on how many of those treatments fail and eventually require prophylactic fixation, the use of ICD-9 78.55 from the beginning may be the most cost-effective approach.
